# Trueness of CAD/CAM digitization with a desktop scanner – an in vitro study

**DOI:** 10.1186/s12903-019-0976-1

**Published:** 2019-12-12

**Authors:** G. Joós-Kovács, B. Vecsei, Sz. Körmendi, V. A. Gyarmathy, J. Borbély, P. Hermann

**Affiliations:** 1 Department of Prosthodontics, Semmelweis University, Szentkirályi u. 47, Budapest, 1088 Hungary; 2 EpiConsult LLC, 8 The Green, STE A, Dover, DE 19901 USA; 3 Johns Hopkins Bloomberg School of Public Health, Baltimore, MD USA

**Keywords:** Digital dentistry, Desktop scanner, Indirect CAD/CAM digitization, Extraoral scanner, Gypsum cast digitization, Accuracy, Trueness, Precision

## Abstract

**Background:**

Desktop scanners are devices for digitization of conventional impressions or gypsum casts by indirect Computer-Aided Design/Computer-Assisted Manufacturing (CAD/CAM) in dentistry. The purpose of this in vitro study was: 1, to investigate whether virtual models produced by the extraoral scanner have the same trueness as sectioned casts; and 2, to assess if digitization with an extraoral scanner influences the surface information.

**Methods:**

A polimethyl-methacrilic acid (PMMA) cast and a reference scanner (TwoCam 3D, SCAN technology A/S, Ringsted, Denmark; field of view 200 mm, resolution 0.1 mm ± 0.025 mm) were used to create the reference data in standard tessellation format (STL). According to the extraoral CAD/CAM digitization steps, impressions, mastercasts, and sectioned casts were made, and STL files were generated with the reference scanner. The pivotal point of the study was to digitalize these sectioned casts with the extraoral scanner (Straumann CARES Scan CS2 Visual 8.0 software, InstitutStraumann AG, Basel, Switzerland) and STL files were exported. Virtual caliper measurements were performed. Absolute deviations were compared using multilevel mixed-effects linear regression. Relative distortions were calculated with mean absolute errors and reference values.

**Results:**

Differences were observed in measurements of tooth sizes. All four prepared teeth were affected. No relationship was observed in relative deviations. Absolute differences between all the indirect digitization steps considering arch distances were: impressions, − 0.004 mm; mastercasts, 0.136 mm; sectioned casts, − 0.028 mm; and extraoral scanner, − 0.089 mm. Prepared dies on the virtual casts (extraoral scanner) were closer to each other than those on the sectioned gypsum casts. Relative deviation calculations revealed no relationship with the position of the dies in the arch.

**Conclusion:**

The trueness of the virtual models generated by the extraoral scanner system used in this study was different from the dimensions of the sectioned casts. The digitization of gypsum casts changes both the dimensions of dies and the distances between the dies. The virtual casts had smaller distances than any distances measured at previous steps. Either bigger dies or longer distances did not result in greater distortions. We cannot, however, generalize our results to all scanners available on the market, because they might give different results.

## Background

Digital technology is nowadays essential in many aspects of life including industry, social life, entertainment, and health care as well. For example, digital dental technology (DDT) offers quicker, better solutions to patients: X-ray [1], CBCT [1], and digital tooth shade determination devices [2] help to improve diagnosis and treatment plan. Extra- and intraoral scanners are also widely used to make fixed dental restorations, which promise not only better accuracy to the final rehabilitation but also better time efficiency and comfort [3–5]. DDT has also been introduced in education, and students also seem to prefer the optical impression taking method compared to the conventional one [5, 6].

Computer-Aided Design/Computer-Assisted Manufacturing (CAD/CAM) systems promise the opportunity to improve accuracy by reducing potential sources of error (such as, for example, the lost-wax process) [7–10]. As the first step, a scanner (either extraoral or intraoral) is utilized to transfer the information of the oral cavity into the computer. Extraorally, indirect CAD/CAM digitization is usually performed by scanning gypsum casts, although sometimes conventional impressions may also be scanned [7, 11]. The intraoral scanning technique performs a direct scanning in the oral cavity [8, 12]. Some studies assess the patients’ perception and treatment comfort, clinical outcomes, and time efficiency [5, 13], while others compare the clinical accuracy of conventional impressions with that of direct digitizers [9, 14, 15] or the accuracy of direct versus indirect digitizers [16–18].

To ensure accurate fixed dental restorations, either conventionally or digitally made, inaccuracies during the entire process should be minimized [19–22]. The distortion factors of indirect digitization are well explored according to the conventional impression techniques, impression materials, pouring techniques, gypsum materials and sectioning systems [10, 12, 23–33]. However, the last step of indirect digitization, which is performed with an extraoral laboratory scanner, still leaves lots of questions unanswered. Several studies have assessed extraoral scanners [34–37], but still little is known about their accuracy in actual clinical settings.

The accuracy measurement based on the ISO (International Organization for Standardization) standard 5725 [14, 16–18] has two components: precision and trueness. In a previous study, our research group compared the accuracy of three intraoral scanners to a desktop scanner [18]. One of the results was that the desktop scanner was less accurate compared to the intraoral scanners. This raised a question: which step or steps of the indirect CAD/CAM digitization changed the original surface information? The aim of this study, therefore, was to evaluate the trueness of virtual models produced by the extraoral scanner. Our hypothesis was that there is no significant difference between the sectioned casts and the virtual casts made by the extraoral scanner.

## Methods

### Study design

**Fig. 1:**
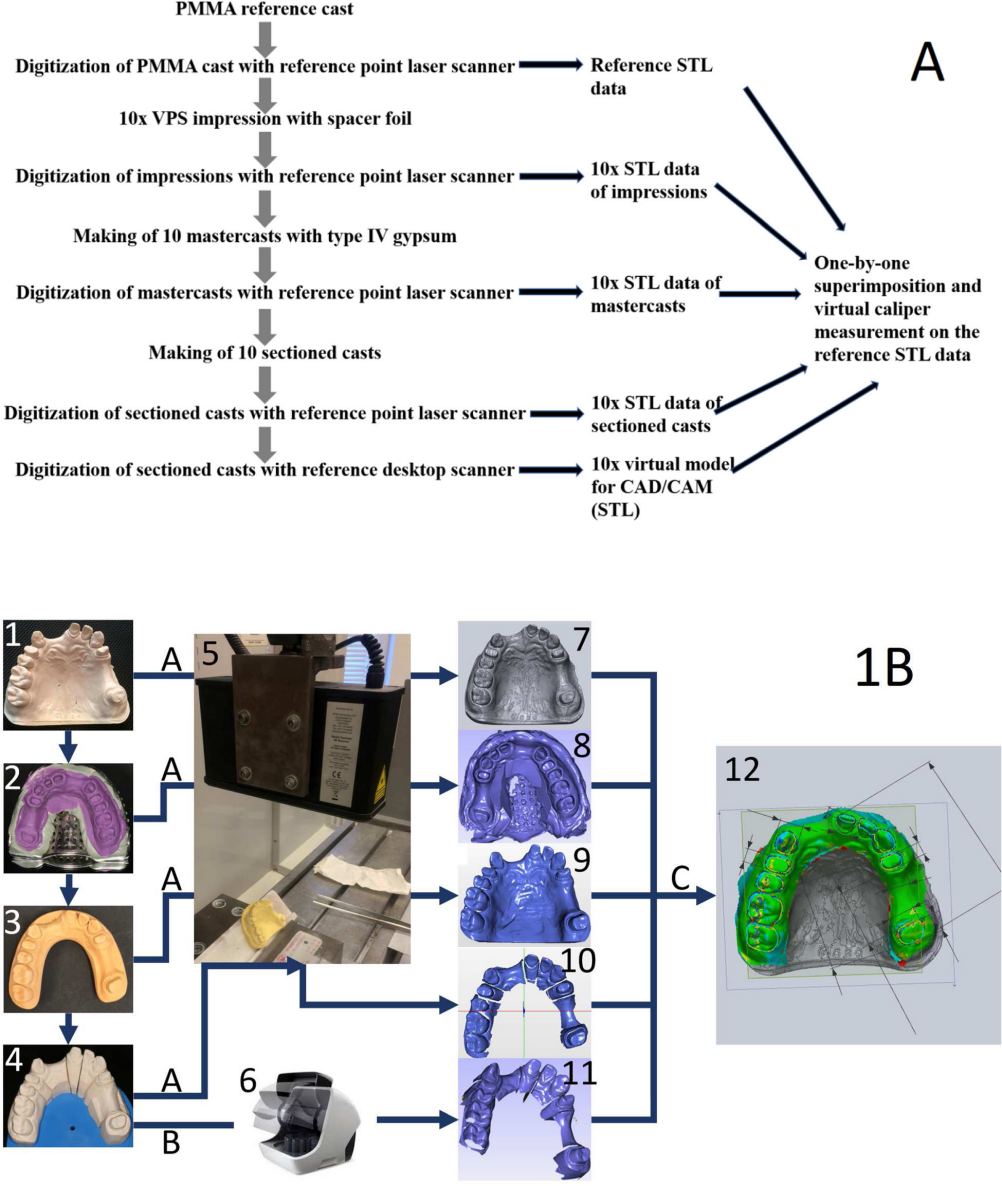
**a** Flowchart depicting the steps of indirect CAD/CAM digitization, **b** Infographic depicting the steps of indirect CAD/CAM digitization

Figures 1a and b depict the study design. A polimethyl-methacrilic acid (PMMA) cast and a reference scanner (TwoCam 3D, SCAN technology A/S, Ringsted, Denmark; field of view 200 mm, resolution 0.1 mm ± 0.025 mm) were used to create the reference data in standard tessellation format (STL). According to the extraoral CAD/CAM digitization steps, impressions, mastercasts, and sectioned casts were made, and STL files were generated with the reference scanner. The pivotal point of the study was to digitalize these sectioned casts with the extraoral scanner (Straumann CARES Scan CS2 Visual 8.0 software, Institut Straumann AG, Basel, Switzerland) and STL files were exported. Accordingly, in order to evaluate the information changes from the reference data through the steps of indirect digitization up to the CAD/CAM scan made by the extraoral scanner, we strictly adhered to the steps of indirect CAD/CAM digitization and compared the data of impressions, mastercasts, sectioned casts, and desktop scanner digitization to the reference data.

### Reference model

**Fig. 2:**
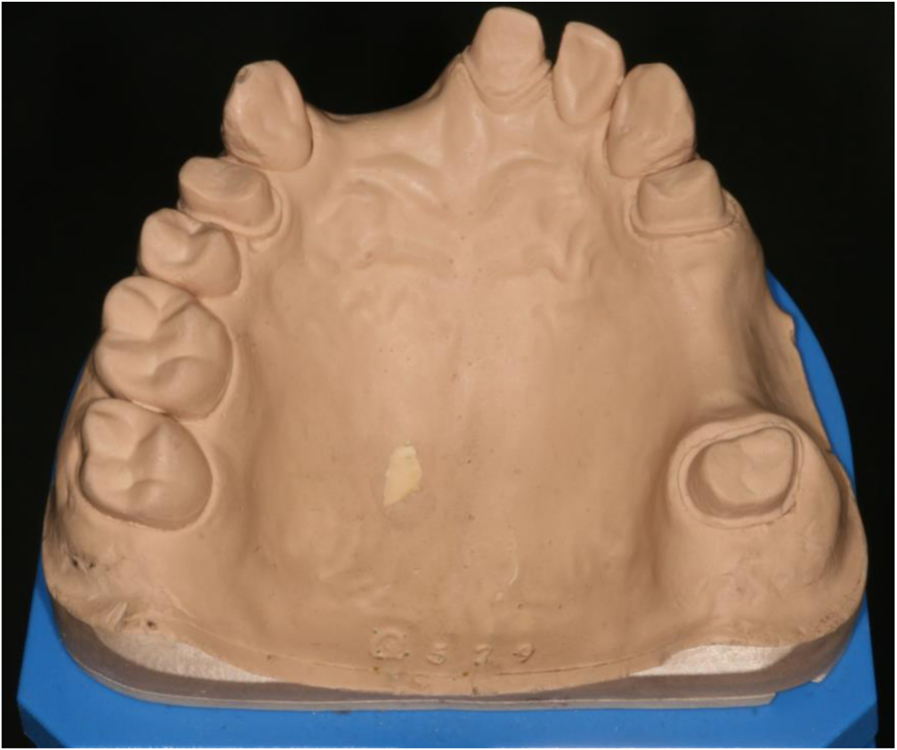
PMMA reference cast. After digitization with the reference scanner, 10 VPS impressions were taken.ű

A PMMA cast was made from an upper arch, holding the original information of hand-prepared teeth #14, #21, #24, #27 with shoulder preparation for all-ceramic restorations. Two edentulous areas are represented on the arch, #13–21 and #24–27 (Fig. 2).

### Reference scanner and reference virtual model

**Fig. 3:**
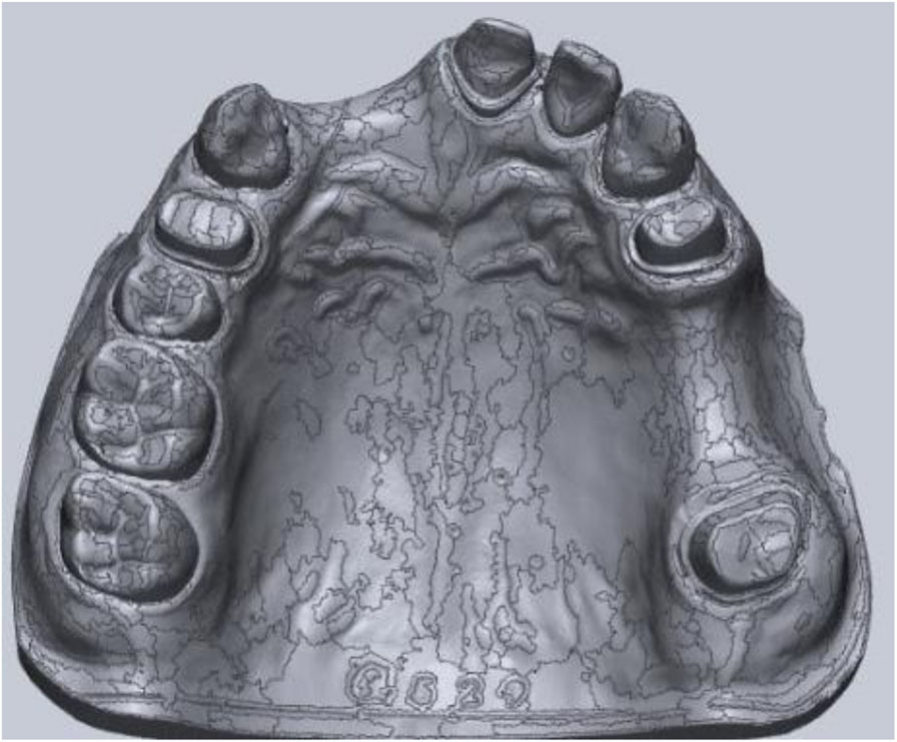
Reference data from the PMMA cast made by the high-precision point-laser scanner

For the high-precision data acquisition, a point-laser scanner (635 nm wavelength, 1 mW power, Class IEC 2) was utilized (TwoCam 3D, SCAN technology A/S, Ringsted, Denmark). This scanner uses a double triangulation technique with the following parameters: field of view 200 mm, resolution 0.1 mm ± 0.025 mm. The reference model was scanned by the point-laser scanner, and an STL reference virtual model was generated (Fig. 3).

### Impression

**Fig. 4:**
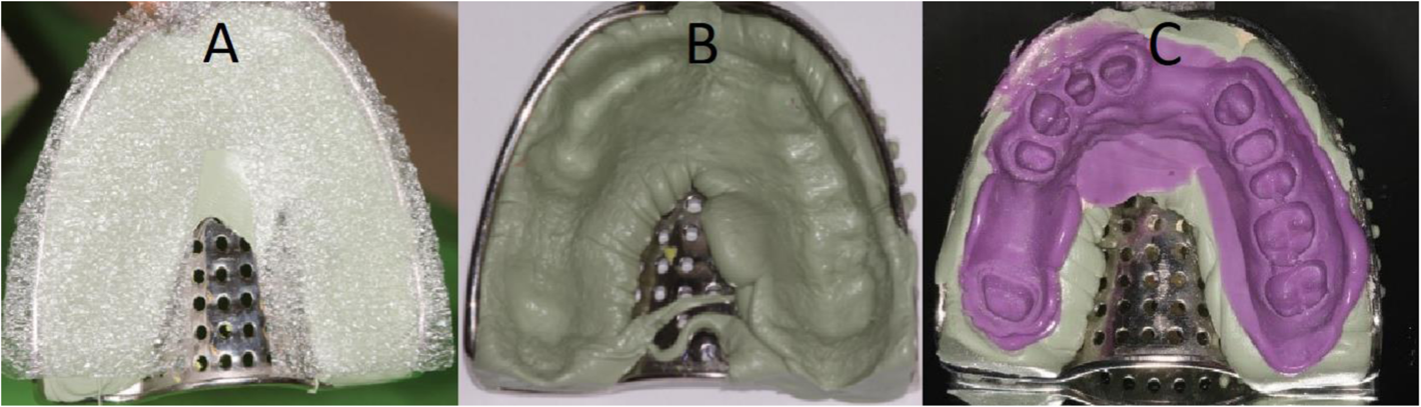
VPS impression with stock metal tray: **a** Putty material with spacer foil; **b** Base impression with putty material; **c** Putty+wash material. Impressions were scanned with the reference scanner at least 1 but not more than 24 h after removing from PMMA cast

Ten VPS (vinyl-polysiloxane impression material, Express XT Penta Putty, Express XT Light Body, 3 M ESPE, St. Paul, MN, USA) impressions were taken with spacer foil technique (Impression Separation Wafer, GC Corporation, Tokyo, Japan), with prefabricated perforated metal tray (Medesy 6000, MEDESY Srl, Maniago, Italy) and machine mixed (Pentamix 3 Automatic Mixing Unit, 3 M ESPE, St. Paul, MN, USA) [10, 23, 24, 31–33] (Fig. 4).

In accordance with prevailing in vitro temperature conditions, the recommended setting time (5 min and 30 s) was doubled to ensure the correct setting of the material [32, 33]. After setting, the impression was removed from the cast and washed for 5 min. Disinfection with Zeta 7 spray (Zhermack, Zhermack Spa, Badia Polesine, Italy) followed. At least 1 h but not more than 24 h after disinfection, each impression was scanned once with the reference scanner – thus, a total of 10 impression scans were performed. The produced STL files were saved.

### Mastercasts and sectioned casts

**Fig. 5:**
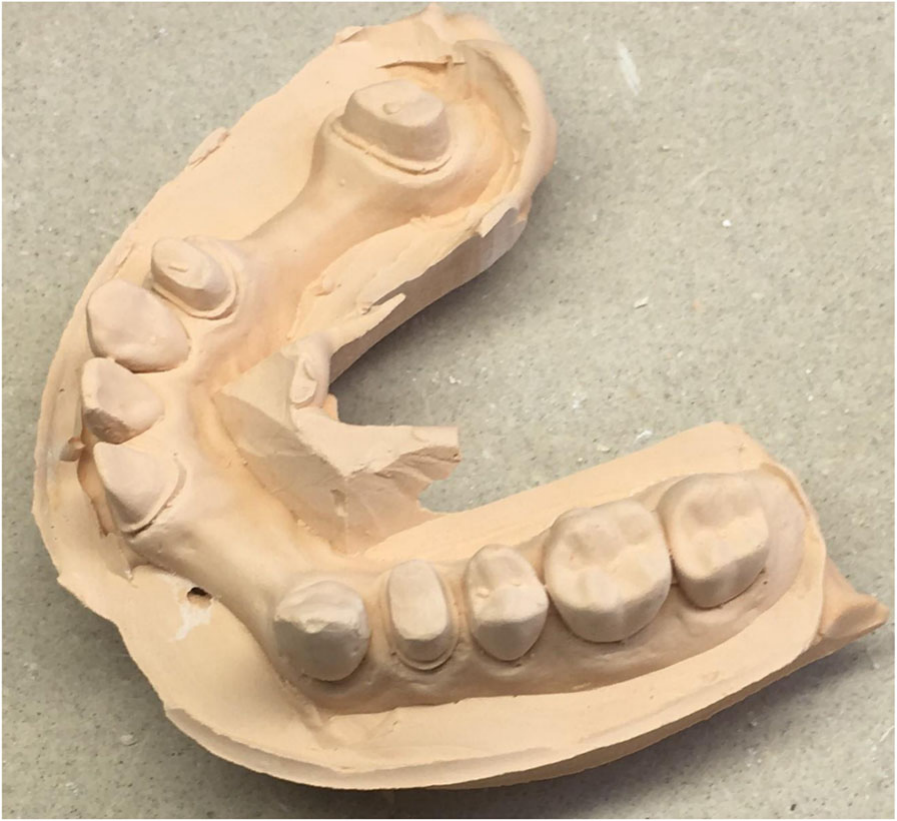
Type IV rough gypsum mastercast after removal of the impression

**Fig. 6:**
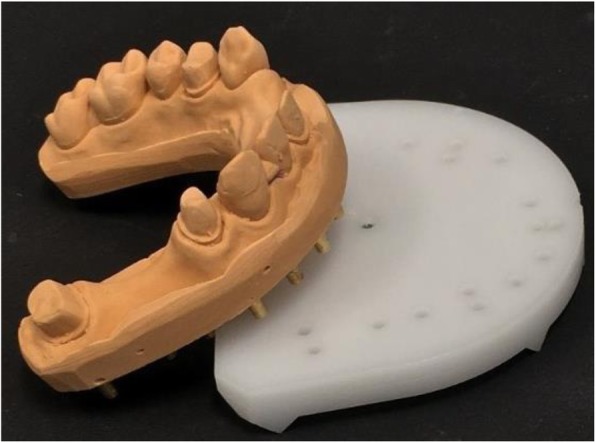
Finalized gypsum mastercasts were scanned with the reference scanner

**Fig. 7:**
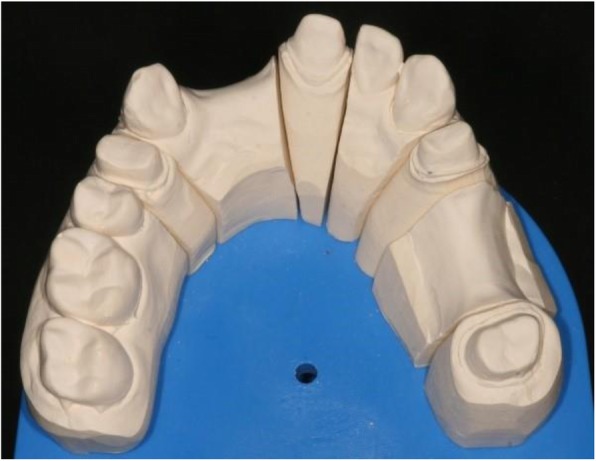
Finalized sectioned cast. Two scannings followed: first, the sectioned casts were digitized with the reference scanner. Second, they were digitized with the desktop scanner

Not more than 24 h after taking the impressions, the 10 impressions were casted in the dental laboratory with type IV gypsum (GC Fujirock EP, GC Corp., Tokyo, Japan) [25, 28, 30]. The mixing of the gypsum was performed with distilled water (100 g/25 ml) first by hand, then with a vacuum mixer (20 s, BEGO Motova SL, BEGO USA Inc., Lincoln, RI, USA). A mechanical vibrator (WASSERMANN Rüttler KV-26, Wassermann Dental-Maschinen GmbH, Hamburg, Germany) was used (6000 rpm, 0.4 mm) to produce the casts. The setting time of the gypsum was always 1 h. After setting, the mastercasts were removed and finalized (Figs. 5 and 6). All 10 mastercasts were digitized with the reference scanner, and the STL files were saved. Next, the mastercasts were sawed in the laboratory (Giroform, Amann Girrbach GmbH, Pforzheim, Germany), and 10 sectioned casts were scanned with reference scanner (Fig. 7).

**Fig. 8:**
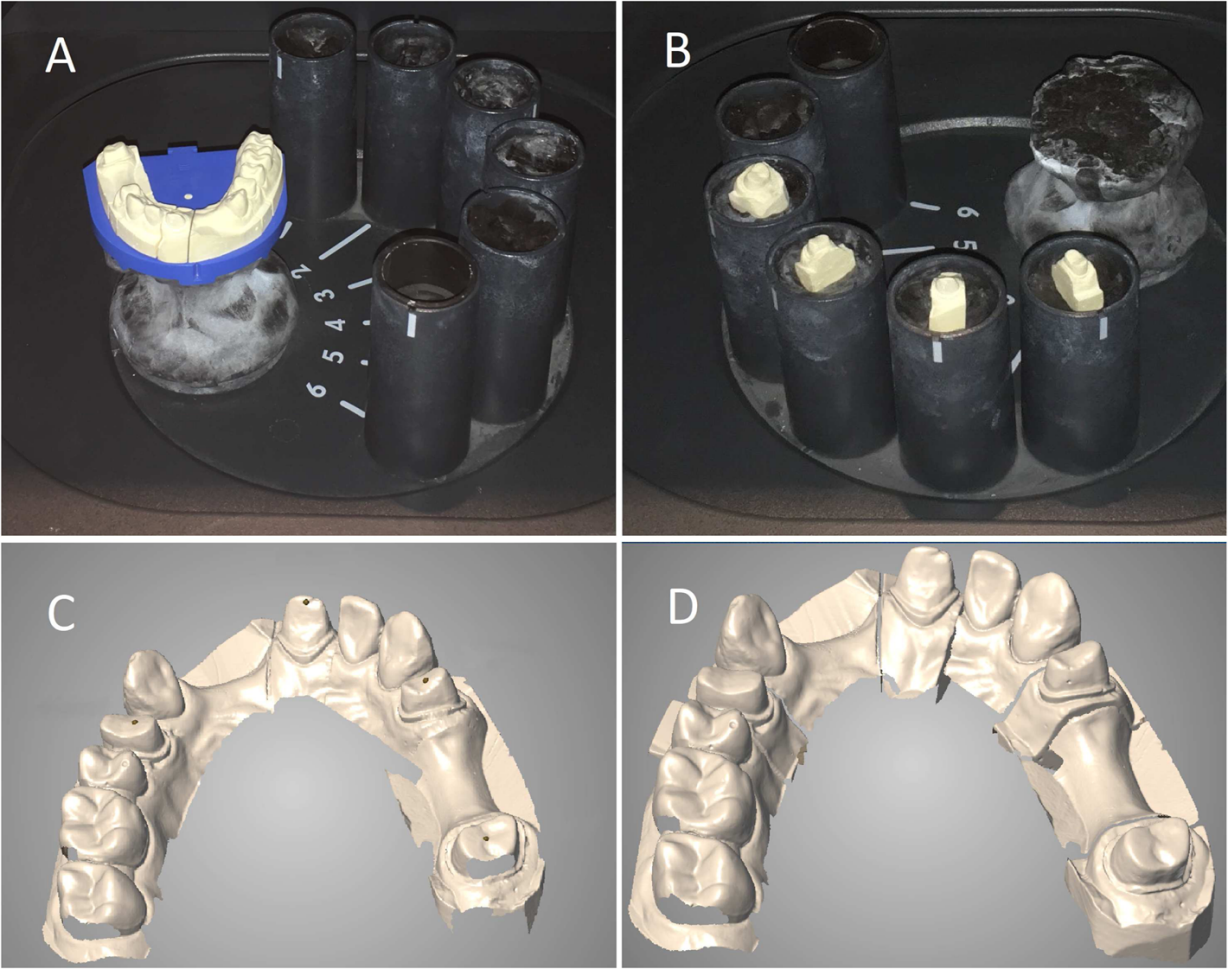
Digitization of sectioned cast with desktop scanner: **a** Full arch gypsum model placed in the desktop scanner to make a whole-arch scan. **b** Removeable gypsum dies in the scanner before second scanning. **c** First scan of the full arch model with gap on the prepared 27 tooth. **d** Final virtual model with the secondary dye scans aligned

To perform CAD/CAM scans used in CAD/CAM technology [12], the sectioned casts were scanned 24–72 h after the casting of the impressions with an extraoral scanner (Straumann CARES Scan CS2 Visual 8.0 software, Institut Straumann AG, Basel, Switzerland) in line with the manufacturer’s instructions (Figs. 7d - 8a). This involves a first full-arch scan of the gypsum cast followed by a second scanning of the prepared dies. The software aligns the die scans onto the full arch scan, and this alignment will lead to higher precision of the scanned data. The STL files of 10 final virtual casts were exported and saved.

### Superimposition and virtual digital caliper

**Fig. 9:**
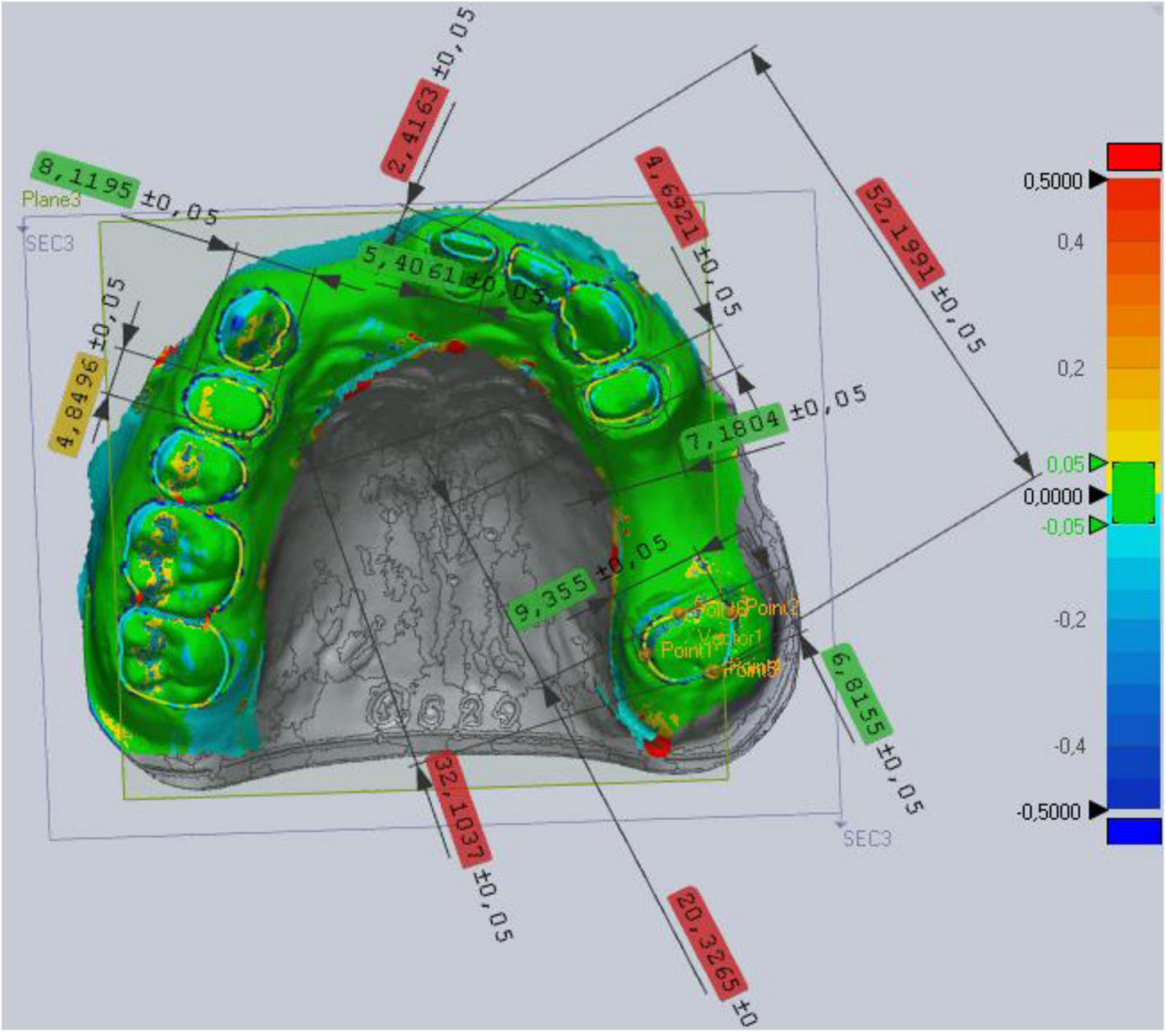
Virtual digital caliper measurements on prepared teeth: between the mesio-distal and bucco-palatinal points of 14, 21, 24, 27 teeth and 3 arch distances: between the closest points of 24–27, furthest points of 24–27, and furthest points of 21–27

For the virtual comparisons, a best-fit alignment algorithm and virtual caliper tool were used in Geomagic Verify software (3D systems, 333 Three D Systems Circle, RockHill, SC, USA). Eleven virtual caliper measurements were performed on the reference virtual model as follows: a section plane was placed on the virtual casts, and on this plane, mesial-distal points and buccal-oral points of the prepared teeth and 6 points for the measurements of abutment distances were appointed (Fig. 9). Each impression, mastercast, and sectioned cast STL file made by the reference scanner and the STL files from the sectioned casts made by the extraoral scanner were imported and aligned one after the other to the virtual reference model by best-fit alignment. The Geomagic software calculated the differences between the distances measured on the reference versus the superimposed data and output the resulting data in Excel.

The following measurements were taken

A. Tooth sizes
  1. 14 mesio-distal (14MD) and bucco-palatinal (14BP) diameter,
  2. 21 mesio-distal (21MD) and bucco-palatinal (21BP) diameter,
  3. 24 mesio-distal (24MD) and bucco-palatinal (24BP) diameter,
  4. 27 mesio-distal (27MD) and bucco-palatinal (27BP) diameter,
B. Arch distances:
  1. closest points of teeth 24–27 as “inside”,
  2. furthest points of teeth 24–27 as “outside”,
  3. furthest points of teeth 21–27 as “left side”.

### Statistics

Observation of differences between the data points was performed in two ways. First, absolute deviations and differences were calculated (in mm). Absolute deviations were compared across CAD/CAM steps using multilevel mixed-effects linear regression and were interpreted as differences in trueness. Explanatory variables included a categorical indicator for each CAD/CAM step and a random intercept term at the observation series level. The model allowed for intragroup correlation between observations within the same observation series. Models were fitted separately for each location. Differences between CAD/CAM steps were expressed as point estimates of the fixed effect, 95% confidence intervals (CI), and *p* values.

Second, relative distortions were calculated. To avoid misrepresentations arising from calculating the averages of positive and negative values (which can result in smaller deviations), absolute values of the observed distances were used. For each die diameter and arch distance, a mean absolute error was calculated. A relative distortion was calculated with mean absolute error and reference value registered with the reference scanner (relative distortion = mean absolute error/reference value). Median and interquartile range values were used because of skewness. Stata was used for data management and analysis (StataCorp. Stata Statistical Software: Release 15. College Station, Texas: StataCorp LLC).

## Results

### Differences in the measurements of tooth sizes

**Fig. 10:**
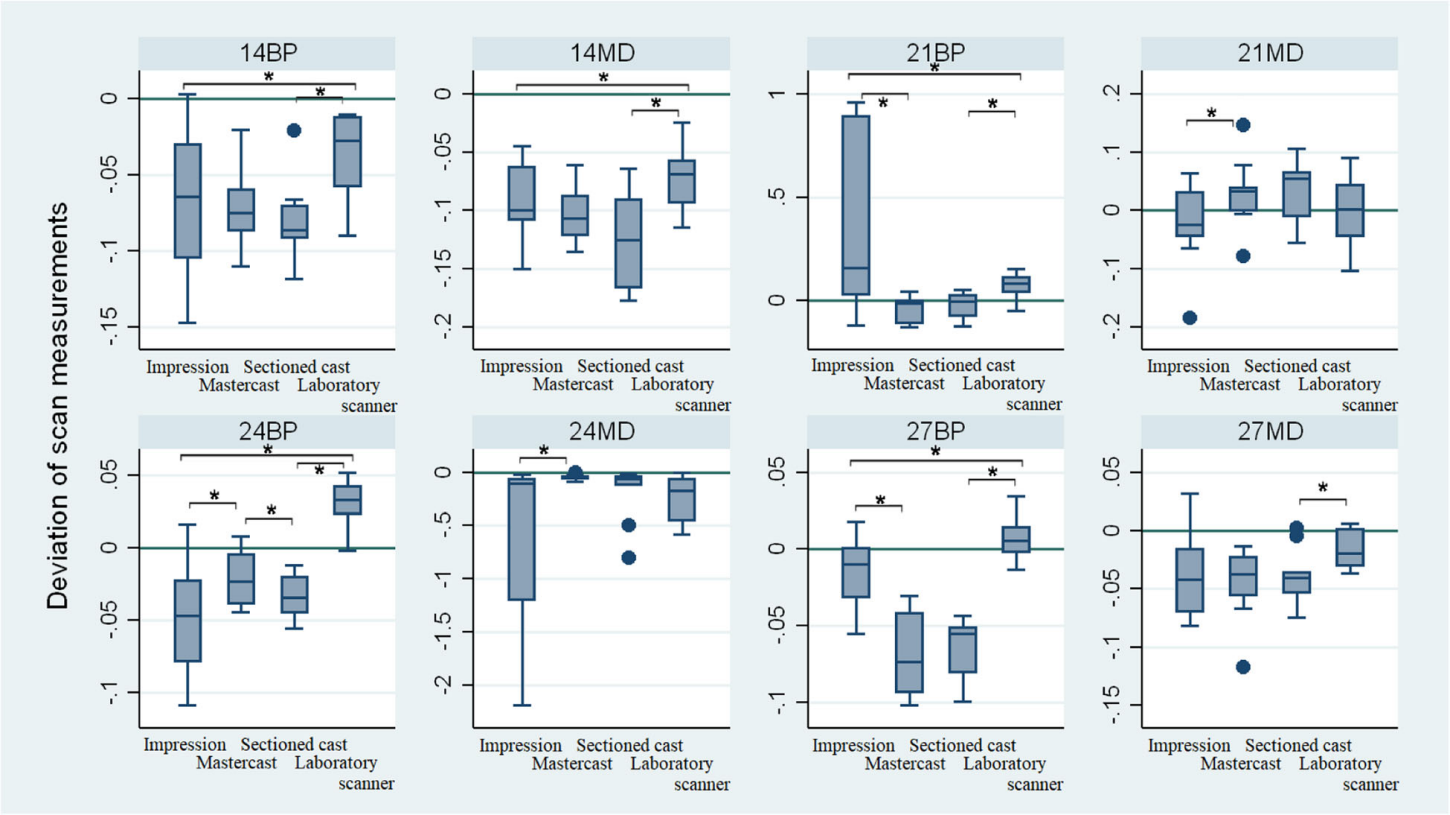
– Absolute distortions of teeth registered by the virtual caliper measurement on the prepared teeth 14, 21, 24, 27. MB and BP distance (mm). Changes are shown by following the indirect CAD/CAM steps (x-axis): 1, Impression; 2, Mastercast; 3, Sectioned cast data gathered with reference scanner (Sectioned cast); 4, Sectioned cast data gathered with desktop scanner (Laboratory scanner)

**Fig. 11:**
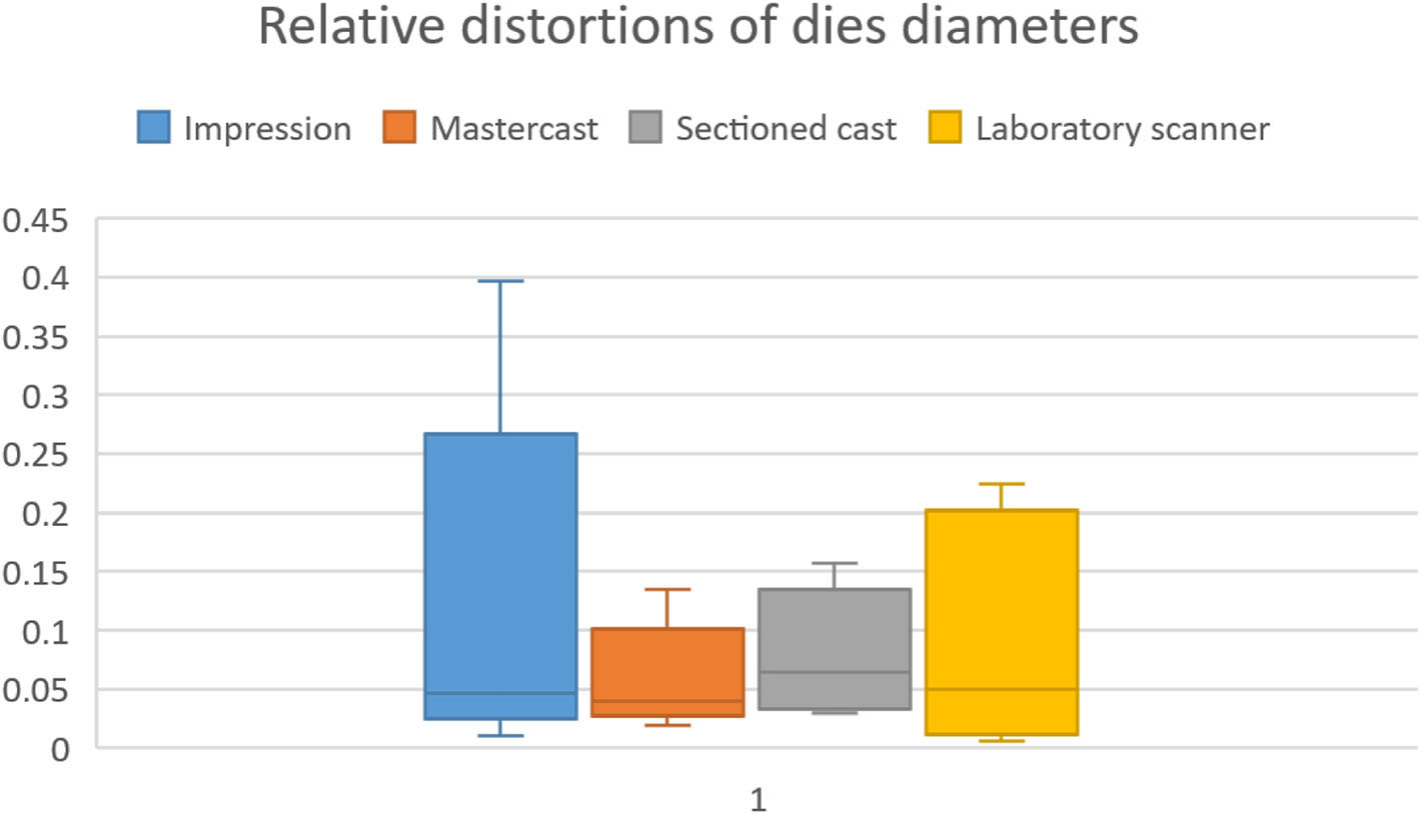
Relative distortions of 8 die diameters were calculated: relative distortion = mean absolute error/reference value. There was no correlation with size. However, teeth in the impressions showed the greatest distortion

Significant differences were observed in the measurements of tooth diameters between the steps of indirect CAD/CAM technology performed with the Straumann extraoral scanner at all eight locations of interest (at a minimum of one and a maximum of four per location) according to the absolute deviations (Fig. 10 and Tables 1 and 2). Overall, the highest number of differences were as follows. Between the sectioned cast and the extraoral scanner, significant differences (either higher or lower) were observed in six of the eight locations (*p* < 0.01), and the data measured on the impressions were significantly different from the data measured on the extraoral scanner at five locations (*p* < 0.05). All four prepared teeth were affected by these differences. There were differences between the steps of indirect CAD/CAM, but no relationship was observed in relative deviations (Fig. 11 and Table 3), meaning that longer diameters did not result in greater distortions.

**Table 1:** MB and BP distance (mm) changes observed by following the indirect CAD/CAM steps

	Q1	Median	Q3	IQR
*14BP*				
Impression	− 0.115	− 0.064	− 0.026	0.089
Mastercast	− 0.092	− 0.075	− 0.058	0.034
Sectioned cast	− 0.095	− 0.086	− 0.069	0.026
Laboratory scanner	− 0.058	− 0.027	− 0.012	0.046
*14MD*				
Impression	−0.118	− 0.099	− 0.061	0.057
Mastercast	−0.124	− 0.107	− 0.085	0.039
Sectioned cast	−0.168	− 0.125	− 0.086	0.082
Laboratory scanner	−0.095	− 0.069	− 0.055	0.040
*21BP*				
Impression	0.026	0.157	0.899	0.873
Mastercast	−0.107	− 0.012	0.004	0.112
Sectioned cast	−0.082	− 0.002	0.032	0.114
Laboratory scanner	0.031	0.083	0.116	0.085
*21MD*				
Impression	−0.049	−0.025	0.039	0.089
Mastercast	−0.002	0.033	0.049	0.051
Sectioned cast	−0.012	0.055	0.067	0.079
Laboratory scanner	−0.048	0.001	0.055	0.103
*24BP*				
Impression	−0.082	− 0.047	−0.019	0.063
Mastercast	−0.041	−0.023	− 0.004	0.036
Sectioned cast	−0.046	−0.034	− 0.019	0.027
Laboratory scanner	0.021	0.033	0.043	0.022
*24MD*				
Impression	−1.317	−0.108	−0.065	1.253
Mastercast	−0.063	−0.044	− 0.032	0.031
Sectioned cast	−0.217	− 0.066	− 0.035	0.183
Laboratory scanner	−0.466	− 0.173	− 0.056	0.410
*27BP*				
Impression	−0.033	−0.009	0.002	0.035
Mastercast	−0.094	− 0.073	− 0.039	0.055
Sectioned cast	− 0.084	− 0.055	− 0.049	0.034
Laboratory scanner	−0.004	0.005	0.015	0.019
*27MD*				
Impression	−0.072	− 0.042	− 0.004	0.068
Mastercast	−0.058	−0.037	− 0.021	0.038
Sectioned cast	−0.058	− 0.041	− 0.028	0.030
Laboratory scanner	−0.031	− 0.019	0.002	0.033

**Table 2:** Significance levels of die diameters according to multilevel mixed-effect linear regression

	95%CI	p
*14BP*		
Impression vs Mastercast	−0.031, 0.025	0.850
Mastercast vs Sectioned cast	−0.016, 0.002	0.103
Sectioned cast vs Laboratory scanner	0.032, 0.063	< 0.001*
Impression vs Laboratory scanner	0.003, 0.071	0.032*
*14MD*		
Impression vs Mastercast	−0.027, 0.009	0.311
Mastercast vs Sectioned cast	−0.055, 0.009	0.152
Sectioned cast vs Laboratory scanner	0.028, 0.084	< 0.001*
Impression vs Laboratory scanner	0.008, 0.040	0.004*
*21BP*		
Impression vs Mastercast	−0.702, − 0.168	0.001*
Mastercast vs Sectioned cast	− 0.020, 0.050	0.411
Sectioned cast vs Laboratory scanner	0.066, 0.119	< 0.001*
Impression vs Laboratory scanner	−0.592, − 0.063	0.015*
*21MD*		
Impression vs Mastercast	0.024, 0.079	< 0.001*
Mastercast vs Sectioned cast	−0.032, 0.044	0.747
Sectioned cast vs Laboratory scanner	−0.069, 0.001	0.060
Impression vs Laboratory scanner	−0.009, 0.057	0.152
*24BP*		
Impression vs Mastercast	0.006, 0.047	0.011*
Mastercast vs Sectioned cast	−0.019, − 0.004	0.002 *
Sectioned cast vs Laboratory scanner	0.056, 0.072	< 0.001*
Impression vs Laboratory scanner	0.055, 0.103	< 0.001*
*24MD*		
Impression vs Mastercast	0.024, 1.024	0.040 *
Mastercast vs Sectioned cast	−0.288, 0.027	0.104
Sectioned cast vs Laboratory scanner	−0.297, 0.174	0.610
Impression vs Laboratory scanner	−0.051, 0.715	0.089
*27BP*		
Impression vs Mastercast	−0.064, − 0.042	< 0.001*
Mastercast vs Sectioned cast	−0.007, 0.014	0.487
Sectioned cast vs Laboratory scanner	0.056, 0.083	< 0.001*
Impression vs Laboratory scanner	0.010, 0.031	< 0.001*
*27MD*		
Impression vs Mastercast	−0.048, 0.031	0.663
Mastercast vs Sectioned cast	−0.017, 0.024	0.735
Sectioned cast vs Laboratory scanner	0.008, 0.041	0.004*
Impression vs Laboratory scanner	−0.013, 0.051	0.248

Note: significant values are marked with *

**Table 3:** Relative calculated distortions of 8 die diameters

	Q1	Median	Q3	IQR
Impression	0.038	0.046	0.136	0.098
Mastercast	0.035	0.039	0.068	0.033
Sectioned cast	0.036	0.064	0.111	0.075
Laboratory scanner	0.018	0.049	0.178	0.160

### Differences in the measurements of arch distances

**Fig. 12:**
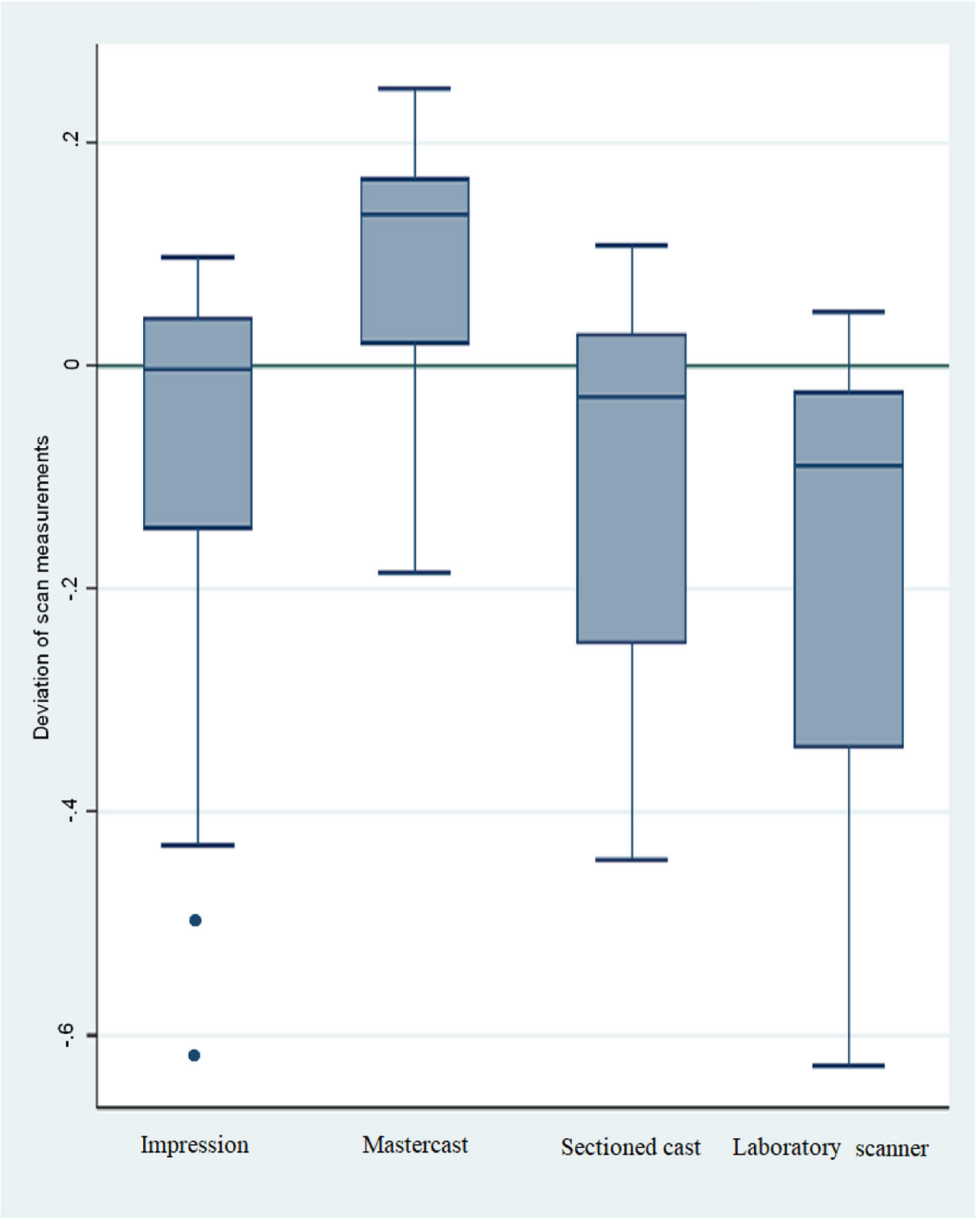
– Observed absolute distortions on all three distances combined (mm) (Inside + outside + left side) The smallest values were obtained from the virtual models generated in the desktop scanner

Statistically significant differences were observed between all the indirect CAD/CAM digitization steps considering arch distances (Table 6). The distance between the dies became larger after the impressions (median − 0.004 mm, IQR = 0.198) were poured with gypsum and the mastercasts (0.136 mm, IQR = 0.157) were made. By sectioning the mastercast, the distances became smaller and the values of the sectioned casts (− 0.028 mm, IQR = 0.279) were similar to those of the impressions. Virtual casts (− 0.089 mm, IQR = 0.322) made by the extraoral scanner showed smaller distances compared to any of the previous steps’ values (Fig. 12 and Table 4).

**Table 4:** Observed distortion on all three distances (mm) (Inside + outside + left side)

	Q1	Median	Q3	IQR
Impression	−0.156	−0.004	0.043	0.198
Mastercast	0.011	0.136	0.168	0.157
Sectioned cast	−0.250	−0.029	0.028	0.279
Laboratory scanner	−0.347	−0.089	− 0.024	0.322

### 24–27 inside

Distance measurement on the closest points of 24–27 showed the following: VPS impressions had the trueness of 0.006 mm (IQR = 0.071), while mastercasts had 0.149 (IQR = 0.034) and sectioned casts had − 0.023 mm (IQR = 0.073). Virtual casts made by the extraoral scanner had the trueness of − 0.086 mm (IQR = 0.043).

### 24–27 outside

Distance measurements on the furthest points of 24–27 showed 0.038 mm (IQR = 0.051) at the VPS impressions. Mastercasts had a trueness of 0.177 mm (IQR = 0.093) and sectioned casts had 0.037 mm (IQR = 0.075). The extraoral scanner-made virtual casts had the only negative value at this distance: − 0.006 mm (IQR = 0.103).

### 21–27 left side

Distance measurement on the furthest points of 21–27 showed the greatest distortions: the VPS impressions had the value of − 0.240 mm (IQR = 0.306). The mastercasts were the closest to the reference value: − 0.050 mm (IQR = 0.13). The sectioned casts were the closest to the impression value at − 0.276 mm (IQR = 0.121). Virtual casts made by the extraoral scanner had a trueness of − 0.398 mm (IQR = 0.169).

The analysis revealed significant distortion in trueness at all steps of the indirect CAD/CAM method, and significant differences were found between the impressions and virtual casts considering all three measured distances (*p* < 0.01, Table 6). The smallest values were registered on the virtual casts made by the extraoral scanner, which were significantly smaller than the values of the impressions and the sectioned casts.

**Fig. 13:**
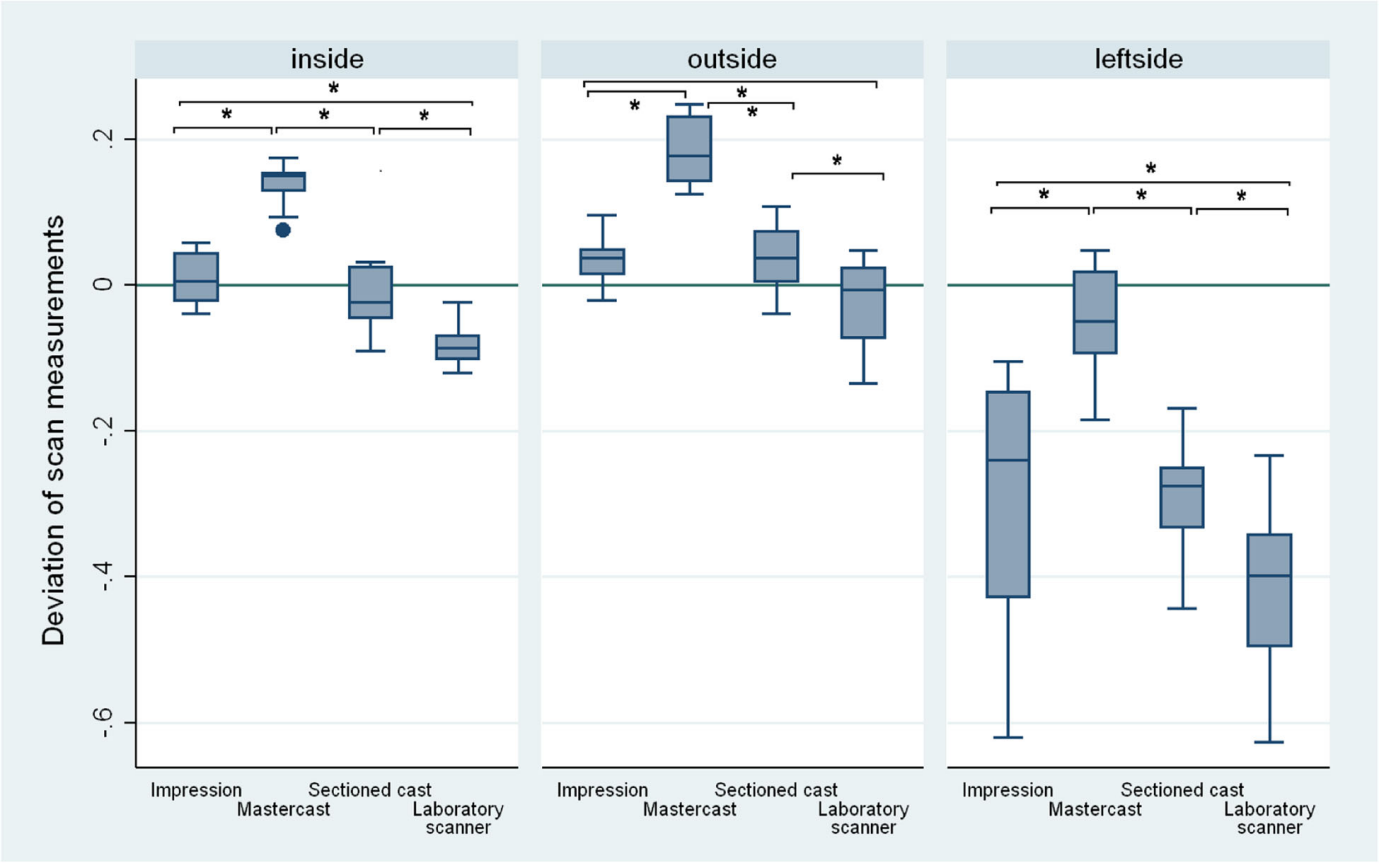
Inside (closest points of 24–27 teeth), outside (furthest points of 24–27 teeth) and left side (furthest points of 21–27 teeth) absolute distance changes (mm) observed at the indirect CAD/CAM steps. The biggest distortions are represented between teeth 21 and 27

**Fig. 14:**
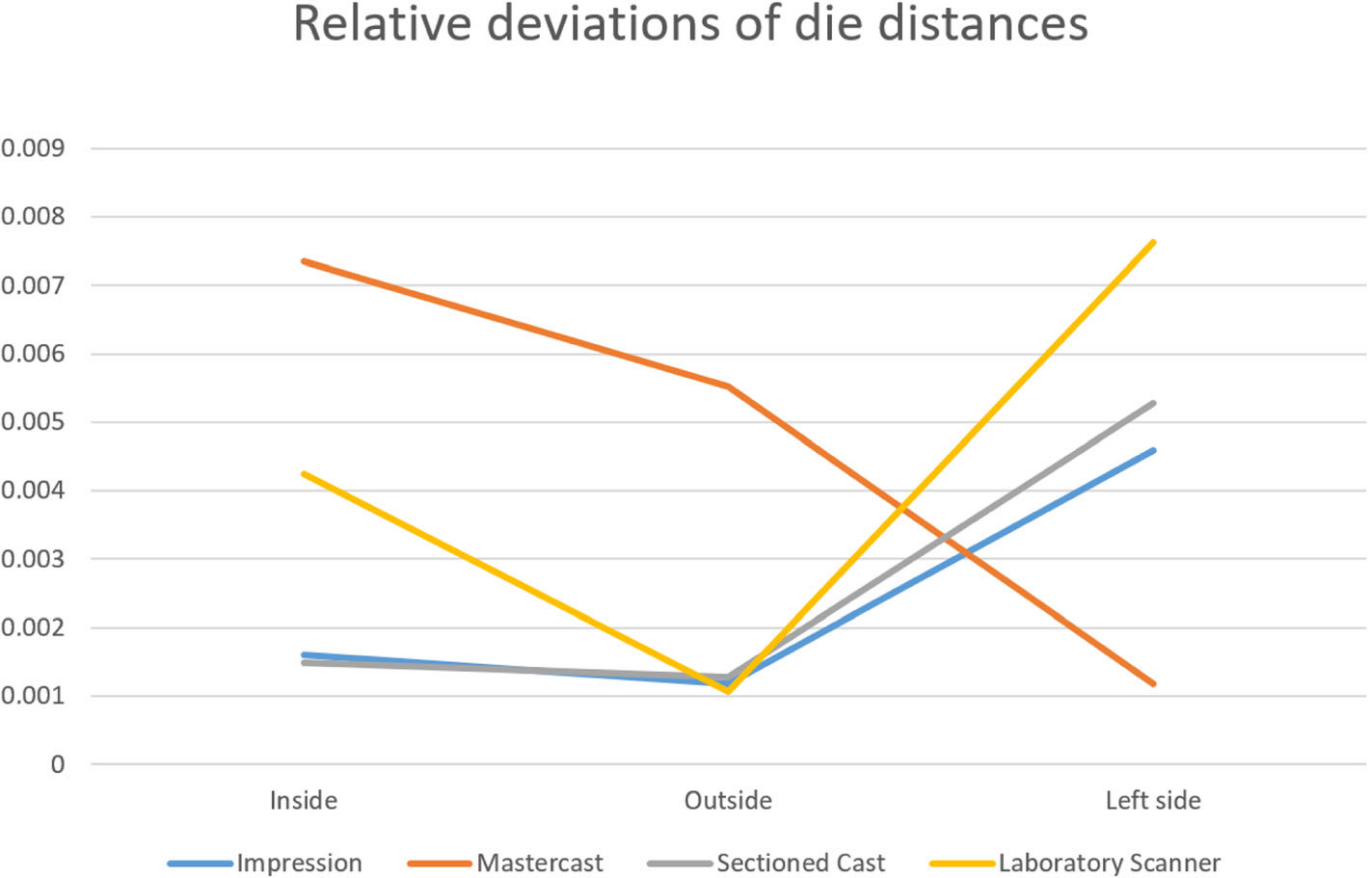
Non-linear relative deviations according to the position of dies in the arch. The smallest relative distortions were observed at middle distance at 3 out of 4 steps (Impressions, sectioned casts data made by reference scanner and sectioned casts data made by desktop scanner)

Overall, absolute deviation calculations showed that the dies on the virtual casts made by the extraoral scanner were larger than those on the sectioned gypsum casts. However, prepared dies were closer to each other on the virtual casts made by the extraoral scanner compared to the sectioned casts made by the reference scanner (Fig. 13 and Table 5). Relative deviation calculations revealed no relationship with the position of dies in the arch (Fig. 14 and Table 6 and 7), meaning that further distances did not result in higher distortions.

**Table 5:** Inside, outside and left side changes (mm) observed at the indirect CAD/CAM steps

	Q1	Median	Q3	IQR
*Inside*				
Impression	−0.023	0.006	0.048	0.071
Mastercast	0.120	0.149	0.154	0.034
Sectioned cast	−0.048	−0.023	0.026	0.073
Laboratory scanner	−0.105	−0.086	− 0.062	0.043
*Outside*				
Impression	0.010	0.038	0.061	0.051
Mastercast	0.139	0.177	0.232	0.093
Sectioned cast	0.004	0.037	0.080	0.075
Laboratory scanner	−0.075	−0.006	0.029	0.103
*Left side*				
Impression	−0.447	−0.24	− 0.140	0.306
Mastercast	−0.105	−0.050	0.026	0.130
Sectioned cast	−0.355	−0.276	− 0.234	0.121
Laboratory scanner	−0.498	−0.398	− 0.329	0.169

**Table 6:** Comparison of the steps of indirect CAD/CAM method on arch distances

	95%CI	p
*Inside*		
Impression vs Mastercast	0.109, 0.141	< 0.001
Mastercast vs Sectioned cast	− 0.186, − 0.13	< 0.001
Sectioned cast vs Laboratory scanner	− 0.083, − 0.038	< 0.001
Impression vs Laboratory scanner	− 0.118, − 0.069	< 0.001
*Outside*		
Impression vs Mastercast	0.103, 0.159	< 0.001
Mastercast vs Sectioned cast	−0.161, − 0.128	< 0.001
Sectioned cast vs Laboratory scanner	−0.075, − 0.02	< 0.001
Impression vs Laboratory scanner	− 0.094, − 0.027	< 0.001
*Left side*		
Impression vs Mastercast	0.162, 0.312	< 0.001
Mastercast vs Sectioned cast	−0.291, − 0.195	< 0.001
Sectioned cast vs Laboratory scanner	−0.173, − 0.071	< 0.001
Impression vs Laboratory scanner	− 0.208, − 0.047	0.002

**Table 7:** Non-linear relative deviations according to the position of dies in the arch

	Inside	Outside	Left side
Impression	0.002	0.001	0.005
Mastercast	0.007	0.006	0.001
Sectioned cast	0.001	0.001	0.005
Laboratory scanner	0.004	0.001	0.008

## Discussion

Little is known about the scanning step in indirect CAD/CAM digitization, and our analysis is one of the first studies to shed more light on the entire process: not only the impression, mastercast, and sectioned cast, but also the digitization step. Our null hypothesis was rejected: our results show that there are differences between the dimensions represented by the sectioned cast and the virtual cast made by the optical extraoral scanner used in the study. We also showed that the scanning procedure influences the diameter of the dies and that there is no relationship between the distance of the dies and the distortion in the measurement of the arch.

Accurate virtual models are necessary to make accurate prostheses. Usually, indirect CAD/CAM digitization is based on conventional impression-taking and stone-cast making procedure. Residual stresses are well known as a feature of gypsum crystallization. ADA Specification #25 describes that the expansion of the dental gypsum might be as much as 0.2% [38]. This distortion caused by expansion can be compensated for by sectioning the cast. In this case, the dies must be in the correct position in the arch, and they must be able to be removed [27]. Sectioning can release stress in the gypsum so that the dies can be repositioned to the original position granted by the acrylic base [30]. The distortion of the conventional cast making steps represented in this study are similar to what has been found in other studies [25, 28, 30]. There have been studies that evaluate the trueness and precision of extraoral scanners by using solo abutments [35], silicone impression material [11], or by an easy-to-measure metric cast [34], but there is hardly any information about the factors that influence accuracy between the making of the gypsum cast and the CAD/CAM production of the final prostheses using life-like samples. This current study provides information about this very important issue.

Our study aimed to assess the trueness of indirect digitization of gypsum casts using an optical extraoral scanner, whereby we hypothesized that there was no difference between the sectioned casts and the virtual casts made by the extraoral scanner. However, when we explored the differences between the trueness of sectioned gypsum cast and the virtual cast made by the optical extraoral scanner, our null hypothesis was rejected. Vanderweghe et al. [34] described how gypsum casts are harder to digitize with optical scanners because of the rough surface. In their study, three out of four scanners had better trueness at scanning acrylic resin. Based on this, the sensitivity of the scanner might also play an important role during scanning. On the other hand, studies show that there is no correlation between triangle numbers (number of digital points) and accuracy, but accuracy depends on the quality of the point cloud generated by the software algorithm [35, 39]**.**

The diameters of the dies were influenced (distorted) by the extraoral scanner in our study. The differences observed on all four prepared teeth #11, #14, #24, #27 during the extraoral scanner scanning step indicates a necessity of caution during everyday dental practice. Our observed distortions, however, differ from those detected in other studies. Wöstmann et al. [40] compared the accuracy of four intraoral and 10 extraoral digitizers in their study, with the reference model die shaping a chamfer-prepared canine and a chamfer-prepared molar. They found differences between the accuracy of intraoral and extraoral scanners with an accuracy below 20 μm. Jeon et al. [41] measured accuracy during extraoral scanning of impressions from prepared teeth for all-ceramic restorations. Their results showed the trueness to be less than 30 μm. The reason for this difference in data could be that these studies measured single prepared teeth only, while in our study two-step scanning procedures were performed on gypsum casts to obtain the whole arch and the dies information as well. However, these differences between the results of the present study and those of other studies are not clinically significant.

The virtual casts made by the laboratory scanner that we used showed the smallest observed values in the arch distances in the whole indirect CAD/CAM digitization process in our study. The distortions observed at small and medium distances have no clinical relevance. The distortions observed at the longest distance (half arch) may have clinical relevance due to their extent (difference between the mean values of the impressions and virtual casts: − 0.158 mm). The relative deviations did not show a relationship with the distortion of the arch. The extraoral scanner used in this study recommends a two-step digitization, and this two-step scanning and the alignment of the data might explain the observed random distortion. Vandeweghe et al. [34] evaluated the accuracy of four different extraoral scanners (Imetric D104i, Imetric 3D; KaVo Everest, KaVo Dental; Smart Optics Activity 880, Smart Optics; Lava ST,3 M ESPE). They used a geometrical model with rectangular and cylindrical shapes as a reference model, and the extraoral scanners were tested with acrylic based casts (mean trueness: 0.047 mm) and gypsum casts (mean trueness: 0.099 mm). Most of their scans met the requirements of clinical accuracy published in other studies [20–22], indicating a lack of clinically unacceptable distortion caused by indirect CAD/CAM digitization. Their results are somewhat different from the results of our present study (our mean trueness: − 0.086 mm). These differences may have arisen from the fact that the two studies used different methods: in addition to us using different casts, the reference model of our study was a PMMA replica of hand-prepared anatomical teeth. As such, the anatomical arch and the prepared teeth in our study may be more difficult for the extraoral scanner to scan that the cylindrical shape in their study.

One limitation of our study is that the trueness in our study might be considered low. Mandelli et al. [35] utilized seven extraoral scanners, although not the Straumann CARES Scan CS2 that we used for scanning, and most of their scanners used only a single-step scanning procedure, while we used a two-step scanning procedure. Furthermore, their reference die was an easy-to-scan solo non-anatomical titan abutment, as opposed to an anatomical gypsum cast with hand prepared abutments used in our study. Their accuracy varied between 8 and 30 μm, depending on the scanner system while our trueness values were much higher. The two-step scanning procedure combined with a hand-prepared abutment with a more complex surface might explain the lower trueness in our study. Another limitation is that our study used only the digitization of a sectioned gypsum cast, and we did not assess the scanning of a precisional-situational impression using a laboratory extraoral scanner or intraoral scanners [7]. However, the scanning of conventional impressions is performed much less often than the scanning of sectioned gypsum casts, and therefore our study focused on the more commonly used indirect digitization. Last but not least, our study focuses only the Straumann extraoral scanner, therefore these results cannot be generalized for all extraoral desktop scanners. However, according to Holst et al. [34] who compared optical and contact scanner accuracy, there is no significant difference between the two types of scanners. A further limitation is that we measured only trueness but not precision – measuring precision might be the topic of future studies. Finally, the laboratory scanner used in this study was not pre-calibrated specifically for the purposes of this study. However, everyday dental laboratory work uses limited calibrations, and therefore our protocol followed a real-life approach.

## Conclusions

Within the limitations of the present in vitro study, we can conclude that the trueness of the virtual models generated by the extraoral scanner system used by us in the study is different compared to the dimensions of the sectioned casts. The digitization of gypsum casts changes both the dimensions of the dies and distances between the dies. The differences observed on all four prepared teeth were both positive and negative at the scanning step. No relationship was observed in relative deviations, meaning that higher values of the dies did not result in higher distortions. At the last step of indirect CAD/CAM digitization, the distances of the virtual casts made by the extraoral scanner were smaller than any of the distances measured at previous steps. No relationship was revealed with the position of dies in the arch, meaning that further distances did not result in greater distortions. Distortions observed at half arch distance may have clinical relevance. We cannot, however, generalize our results to all scanners available on the market, because they might give different results. Therefore, future studies may further explore the accuracy of other extraoral scanners in life-like samples.

## Data Availability

The datasets used and/or analyzed during the current study are available from the corresponding author on reasonable request.

## Abbreviations

BP: Bucco-Palatal diameter of a measured tooth
CAD: Computer-assisted design
CAM: Computer-assisted manufacturing
CBCT: Cone beam computed tomography
DDT: Digital dental technology
MD: Mesio-distal diameter of a measured tooth
PMMA: Polimethyl-methacrilic acid
STL: Standard tessellation format
